# The Effect of Deep Neural Network Implementation on Speech Recognition, Listening Effort, and Sound Quality in Older Adults With Mild to Moderately Severe Hearing Loss

**DOI:** 10.1177/23312165261449983

**Published:** 2026-05-13

**Authors:** Paula Folkeard, Vahid Ashkanichenarlogh, Mohamed Rahme, Vijay Parsa, Volker Kühnel, Bilal Sheikh, Jinyu Qian, Yu-Ying Sung, Susan Scollie

**Affiliations:** 1 National Centre for Audiology, Faculty of Health Sciences, 151162 Western University, London, Canada; 2 Faculty of Health Sciences’ Health and Rehabilitation Program, 151162 Western University, London, Canada; 3 Department of Electrical and Computer Engineering, Western University, London, Canada; 4 Department of Research and Development, 87724 Sonova AG, Stäfa, Switzerland; 5 600267 Department of Research and Development, Sonova Canada Inc., Kitchener, Canada

**Keywords:** deep neural networks, machine learning, denoising, speech-in-noise, listening effort, sound quality

## Abstract

Deep neural network (DNN)-based noise reduction has emerged as a promising advancement in hearing aid signal processing as a means to improve speech intelligibility in noisy environments for hearing aid wearers. The aim of this study was to investigate the impact of the DNN on speech intelligibility in speech-shaped noise (SSN) and multitalker babble (MTB) when the target speech was coming from the front and from the side. Subjective ratings of clarity, total impression, listening effort, and background noise awareness were also collected. Twenty adult participants with mild to moderately severe sensorineural hearing loss were fitted with hearing aids from a single manufacturer, programmed with four different settings that varied across combinations of microphone directionality (omnidirectional and directional beamforming) and noise reduction (off, traditional, DNN). Results showed that DNN, when combined with beamforming, consistently outperformed the other programs across all metrics. Outcomes were influenced by both noise type and spatial configuration. DNN was more effective in SSN than MTB. Beamforming was especially beneficial when the target speech came from the front. Listening in programs that included both DNN and beamforming together resulted in additional benefits shown in the outcome measures, most likely due to the beamforming improving the signal-to-noise ratio and providing a cleaner signal for the DNN to work with.

## Introduction

A current trend in hearing aid manufacturing is the implementation of artificial intelligence to address the most common complaint of hearing aid wearers, difficulty hearing in background noise. Background noise can reduce speech recognition and increase listening effort (Pichora-Fuller et al., 2016). Although the most recent MarkeTrak reports that there continues to be good overall satisfaction with hearing aids (82%), even with advanced digital signal processing, only 70% of hearing aid wearers reported satisfaction with hearing conversation in background noise (Dobyan, 2025). This is consistent with the MarkeTrak 2022 report, where the “ability to minimize background noise” had the highest dissatisfaction rate (Picou, 2022). Methods to improve the signal-to-noise ratio (SNR) for better hearing in background noise have included strategies such as noise reduction and directional microphones with beamforming. For a comprehensive review of these technologies, see Lakshmi et al. (2021) and Lansbergen and Dreschler (2020). A recent special issue (Alexander, 2021) discusses the use of artificial intelligence within hearing aids. Machine learning, which is a division within the umbrella term of artificial intelligence (AI), has been used for more than two decades in hearing aids to classify listening environments and steer the settings of the device within the classification system of automatic programs (Hayes, 2021). Throughout the years, improved applications have allowed for more sophisticated environmental classification and more advanced control of the components, such as the microphone beamformers (Andersen et al., 2021; Fabry & Bhowmik, 2021). In the continued efforts to reduce listening effort and improve speech recognition in noise, the use of AI, including recurrent neural networks (RNNs) and deep neural networks (DNNs), has been studied and is discussed below.

Keshavarzi et al. (2019) reported that signals processed through an applied RNN model, presented over headphones, had improved speech recognition scores and reduced listening effort, particularly in adverse acoustic environments, compared to traditional noise reduction techniques and no signal processing. Fedorov et al. (2020) examined RNN models such as long-short term memory (LSTM) capable of real-time deployment without perceptual loss in audio quality, thus highlighting their potential for use in hearing aids. While RNNs are designed to model temporal dependencies in sequential data, making them effective at capturing the dynamic nature of speech, feedforward DNNs offer advantages in terms of computational efficiency, stability, and suitability for real-time processing. These characteristics make DNNs particularly well suited for hearing aids, where low latency and consistent performance are critical. DNNs have emerged as a powerful paradigm for speech enhancement, particularly in challenging listening environments (Healy et al., 2021). DNNs offer a data-driven solution capable of learning complex mappings between noisy and clean speech. For a review of some key DNN literature up to 2023, see Healy et al. (2023).

One of the earliest applications of DNNs for listeners with hearing loss was proposed by Goehring et al. (2017), who showed that feedforward DNNs could outperform classical noise reduction algorithms in terms of intelligibility for cochlear implant users. More recently, DNN models developed based on generative and speech foundation algorithms (Sutherland et al., 2024) have used the distance between WavLM-based self-supervised speech representations of clean and noisy signals as a perceptually motivated loss function to train a denoising model for hearing aids, resulting in improved recognition and quality metrics without increasing inference complexity. Furthermore, among the DNN algorithms, generative-based speech enhancement architectures have attracted significant attention due to their ability to effectively suppress noise while preserving the perceptual quality and intelligibility of speech (Abdulatif et al., 2024; Ashkani & Parsa, 2024). These methods typically employ a combination of generative and discriminative models to learn complex mappings from noisy to clean speech. These frameworks have emerged as a powerful structure in speech enhancement, especially in applications requiring robust generalization to unseen noise conditions (Shetu et al., 2025). Thus, deep learning has the potential to improve speech intelligibility for individuals with and without hearing loss, particularly in challenging acoustic environments (Thoidis & Goehring, 2024).

Several studies have demonstrated that deep learning algorithms utilizing multiple microphones effectively enhanced speech signals for cochlear implant users in noisy and/or reverberant situations (Gaultier & Goehring, 2024; Kolberg et al., 2025; Saoji et al., 2024). These studies reported substantial improvements in speech intelligibility, with multi-microphone configurations yielding better results than omnidirectional, single-microphone setups or traditional noise reduction strategies.

Diehl et al. (2022, 2023) introduced a real-time deep learning-based denoising system that operated on mobile devices, streaming enhanced audio directly to hearing aids. This system achieved significant improvements in speech-in-noise understanding for hearing aid users, with subjective ratings increasing by over 40% compared to traditional hearing aid processing. The study highlighted the feasibility of integrating advanced deep learning algorithms into portable devices, offering practical solutions for enhancing speech intelligibility in everyday environments. Building on Diehl's work, a DNN-based noise reduction algorithm was implemented directly on Phonak Audéo I90-Sphere receiver-in-the-canal hearing aids. This DNN-based noise reduction system was deployed on a dedicated co-processor clocked at 50 MHz, which operates as part of a speech-processing pipeline designed for highly noisy acoustic environments. The denoising model follows a U-Net architecture and estimates a complex-valued ideal ratio mask across 64 frequency bins using the short-time Fourier transform of the noisy input signal. The enhanced speech produced by the co-processor is subsequently passed to the standard audio processing chain (Hasemann & Krylova, 2024).

Ashkanichenarlogh et al. (2025) evaluated the performance of this implementation across different signal-to-noise ratios, types of background noise, hearing loss severities, and hearing aid venting types in a manikin-based study. Results of that study showed that DNN combined with beamforming outperformed conventional processing across noise types at 0 and +5 dB SNR in moderate reverberation as measured by the Hearing Aid Speech Perception Index.

## The Current Study

Over the past few years, a number of studies have examined the benefits of DNN-based noise reduction implemented in commercial hearing aids (Andersen et al., 2021; Christensen et al., 2024; Kolberg et al., 2025; Saoji et al., 2024). The current study builds upon the manikin-based evaluation by Ashkanichenarlogh et al. (2025), using multiple background noise types and target speech locations to examine outcomes across listening scenarios to evaluate how the DNN denoising processing performs against other noise management technologies. The primary purpose of this behavioral study, however, was to evaluate speech recognition across background noise types and signal azimuths using experienced adult hearing aid wearers with mild to moderately severe sensorineural hearing loss. While digital signal processing techniques may remove the noise, clean the signal, and improve the SNR, they may also introduce distortion of the target signal, and therefore, subjective outcome measures, including ratings of listening effort, background noise awareness, clarity, and total impression, were also collected.

## Methods

This study was approved by the Western University Human Research Ethics Board (ID:126199), and all participants signed a letter of informed consent. Participants were compensated for their time.

### Participants

An a priori power analysis was performed using GPower 3.1 (Faul et al., 2007) based on the results found in Cox et al. (1988) that evaluated speech recognition in noise, with one list pair of the Connected Speech Test (CST) in a similar population of older adults with hearing loss. Using the standard deviation (SD of 5.4) from the condition closest to our measurement condition, the power analysis was completed. The variance was estimated as ±3 SD, with a result of 32.4. This was rounded up to 33 for use within GPower, for an effect with a group mean change of 5%. This generated a suggested minimum sample size of 19 participants. The results of the power analysis suggested that the study was powered at 95% to measure changes of 5% or greater for primary outcomes and for interactions. To confirm this study was sufficiently powered for a linear mixed model analysis, a post hoc power simulation for the linear mixed models was completed with nsim = 1,000 and α = .05, using the simr package (version 1.0.8).

**Figure 1.**
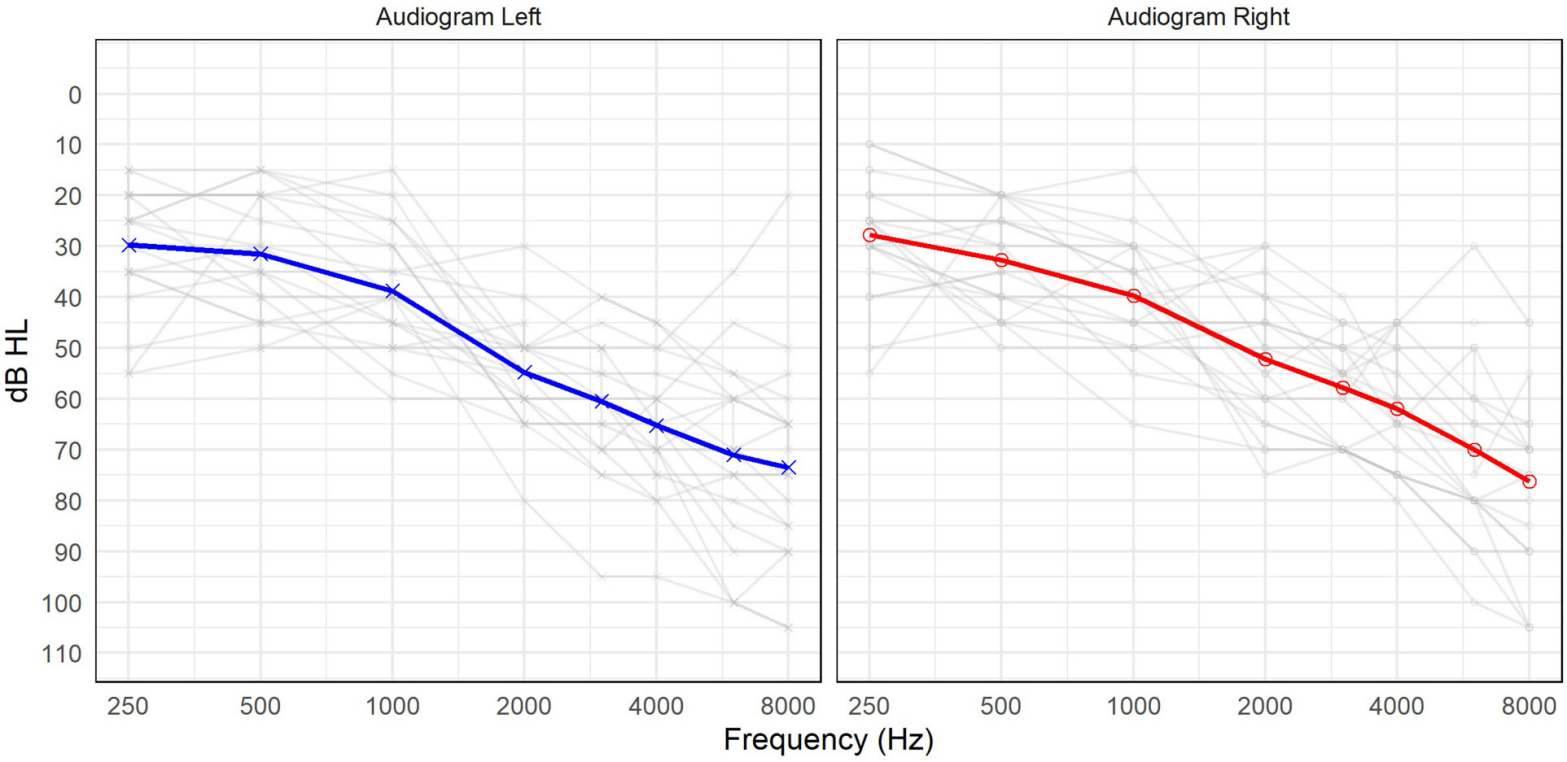
Audiometric thresholds of the participants’ left and right ears (gray). Mean audiometric thresholds of left (blue) and right (red).

Twenty experienced hearing aid wearers with bilateral symmetrical sensorineural hearing loss in the mild to moderately severe range participated. Participants were recruited through the National Centre for Audiology adult participant database (HSREB-110836) and through postings on local community boards and newsletters. All participants were native speakers of English. The four-frequency pure-tone average was calculated per ear to confirm symmetry (<15 dB difference between ears) and to assign a “better ear” for the *Speech from the Side* listening condition described below. Ten participants were women, and 10 were men, with a mean age of 75.6 years (range of 63–86 years). Hearing aid experience ranged from 1 to 35 years, with a mean of 9.8 years. Mean audiometric thresholds are displayed in Figure 1.

### Hearing Aids

Participants were fitted binaurally with commercially available Phonak Audéo I90-Sphere receiver-in-the-canal style hearing aids equipped with M-receivers and domes. The dome style used was based on the recommendation made by the Phonak Target software (version 10.0.1) after the participant's audiogram was entered into the Target software. The hearing aids were fitted to the Adaptive Phonak Digital 3.0 (APD) proprietary algorithm, then the frequency-gain characteristics were adjusted to the Desired Sensation Level v5.0 noise targets (Scollie et al., 2005) at 65 and 75 decibels sound pressure level (dB SPL) using real ear aided response. Individual targets were determined using the Audioscan VF2, the participants measured thresholds, and the individual's real-ear-to-coupler-differences for each ear. The final deviation from target was noted using the default root-mean-square-error function (calculated at 500, 1,000, 2,000, 4,000 Hz) within the Audioscan VF2 (Dao et al., 2021; McCreery et al., 2013). Four manual programs were created using the adjusted frequency-gain characteristics of the fitted main program. Simulated real-ear measures during piloting showed that the activation of the test settings did not affect the frequency-gain response between programs when measured using the International Speech Test Signal (ISTS) in quiet at 65 dB SPL. Table 1 outlines the settings for each of the programs.

**Table 1.** Hearing Aid Settings for the Manual Programs Created for the Test Conditions.

Manual Programs	Test Program Type	Microphone ​	Spheric Speech Clarity ​	Noise ​ Block ​	Description and Processor Names
1	No Processing ​	Omni ​	N/A ​​	Off ​	Omnidirectional microphone with all DSP features off.^a^
2	Beam + Noise Block	Directional ​	N/A ​	Default ​	Speech-In-Loud Noise program with traditional noise reduction (Noise Block) and binaural beamforming (StereoZoom 2.0). All other DSP features off.^a^
3	DNN-only ​	Omni ​	Default ​	N/A ​	Spherical Speech in Loud Noise-only activated. Omnidirectional microphone with all other DSP features off.^a^
4	Beam + DNN	Directional	Default ​	N/A ​	Spherical Speech in Loud Noise and fixed monaural beamforming microphones activated. All other DSP features off.^a^

a Feedback reduction (whistle block) was activated as needed and was held constant across all programs.

### Test Environment

All procedures were completed in a double-walled sound-treated booth at the National Centre for Audiology. Participants were seated in the booth surrounded by 4 Anthony Gallo speakers, placed at 0°, 90°, 180°, 270° azimuth at a distance of 115 cm from the center of the head.

### Test Stimuli

Stimuli from the General North American Accent version of the CST (Saleh et al., 2020; Sung et al., 2025) were presented from a computer using a custom graphical user interface created in MATLAB, routed to a MOTU 12 soundcard, Tucker-Davis Technologies PA5 attenuators, and a QSC amplifier (CX168). This version of the CST comprises 32 passages on common topics (e.g., Clocks, Oranges) spoken by a female talker. Each passage has 9–10 target sentences per topic that include 25 key words that are scored. Participants are told the topic before the passage starts. Continuous background noise, described below, starts 20 s before the first sentence and is played throughout the entire passage.

For this study, CST target passages were played from one speaker (0°) while competing noise was presented from the other three loudspeakers (90°, 180°, 270°). Two types of background noise were used in this study, multitalker babble (MTB) and speech-shaped noise (SSN). The MTB was comprised of three male/female dyads, with one dyad played from each of the three masking loudspeakers. This 2 × 2 × 2 masker resulted in a total of six distinct talkers speaking simultaneously from different directions around the participant. Sung et al. (2025) developed normative performance functions for the 2 × 2 × 2 setup of this CST version. For the SSN condition, an uncorrelated speech-shaped noise, generated to match the overall level and long-term spectral shape of the MTB, was presented from each of the three loudspeakers. The MTB in the 2 × 2 × 2 setup and the SSN were previously used in an objective evaluation of this DNN across different noise types (Ashkanichenarlogh et al., 2025). This behavioral study included both of these noise conditions: MTB was selected to test the processor in a challenging noise type that hearing aid users frequently report is problematic, whereas SSN was included as it allows for high experimental control with no linguistic context, and is frequently used in hearing aid studies to evaluate noise-reduction performance. Speech was presented at 70 dB SPL, and the SNR for both noise types was set to 0 dB. These are challenging but not unrealistic speech and noise levels for hearing aid users (Smeds et al., 2015; Wu et al., 2018).

### Measured Variables

Speech recognition scores were collected for the four different hearing aid settings in the two different noise types, and with speech coming from each of two different azimuths (speech from the front 0° and again from the side that corresponded to the “better ear” (either 90° or 270°)). After each speech recognition condition, subjective ratings of listening effort, background noise awareness, clarity, and total impression were obtained.

### Test Order: Counterbalancing and Randomization

All outcome measures for one azimuth condition were completed before moving to the next azimuth condition. The starting azimuth condition was counterbalanced so that half of the participants started with target speech from the front and half started with speech from the side. Within each azimuth condition, the order for testing for the 16 measures (4 hearing aid settings × 2 noise types × 2 lists per condition) was randomized using the sequence generator in random.org. The researchers changed the hearing aid setting using the Phonak Target programming software so that participants were blinded to which setting was being tested. A unique practice list, not included again for any of the measured variables, was completed at the beginning of each azimuth block using the more difficult MTB and a randomized hearing aid setting.

### Speech in Noise Recognition

#### Speech From the Front

Speech-in-noise recognition scores at 0 dB SNR were collected using the CST in MTB and in SSN. The target speech came from the 0° speaker. The participant faced the 0° speaker, and the maskers were played through the 90°, 180°, and 270° speakers. The participant was told the topic of the passage prior to testing. Two CST lists per hearing aid program were completed, and the keyword correct score was computed out of 50.

#### Speech From the Side

Speech-in-noise recognition scores at 0 dB SNR were collected using the CST in MTB and in SSN. Speech was presented from the 0° speaker. The participant was turned to face either the 90° or the 270° speaker based on their “better ear” 4 frequency puretone average (PTA), or if equal on both sides, the direction was counterbalanced across participants. (This resulted in nine participants turning to face the 90° speaker and 11 participants turning to face the 270° speaker). Speech came from the side, and the maskers came from the other three speakers. Two CST lists per variable were completed for a keyword correct score out of 50.

### Subjective Ratings

After each speech recognition test, the participant rated the clarity and total impression of the target speech they just heard, their awareness of the background noise, and the amount of listening effort they needed to perform the outcome measure. Participants were provided with a sheet of paper that presented a series of Likert scales for reference while completing the ratings. Listening effort and background noise awareness were both rated using a 7-point Likert scale. For listening effort, the participants were asked, “How much effort does it require for you to understand the speech?” A rating of 1 corresponded to “No Effort” and a rating of 7 corresponded to “Extreme Effort” (Johnson et al., 2015). For the background noise awareness rating, participants were asked: “How aware are you of the background noise were you in this setting?” A rating of 1 corresponded to “Not at all aware,” and a rating of 7 corresponded to “Extremely Aware.” As this was rated following each speech-in-noise test, higher ratings on the background noise awareness scale were expected to be driven by the overall noise level and by the extent to which the background noise interfered with the perception of the target speech during the test. With both the listening effort and background noise awareness rating scales, a lower score indicates better performance (i.e., participants rate lower effort and less background noise awareness with lower scores).

The participants were also provided with Gabrielsson Likert scales out of 10 and asked to rate the dimensions of clarity and total impression (Gabrielsson et al., 1988). The Gabrielsson scales range from 0 to 10, with lower scores indicating a poorer rating (i.e., participants rated better clarity and total impression with higher scores).

## Results

### Hearing Aid Fittings

Three participants were fitted with open domes, ten were fitted with vented domes, and seven were fitted with power domes. Participants wore the same dome style in both ears. Mean root mean square error (RMSE) from the Desired Sensation Level (DSL v5.0) noise target (500–4,000 Hz) was calculated at 3.9 dB (SD: 2.3) for 65 dB SPL and at 3.8 dB (SD: 2.3) for 75 dB SPL.

### Statistical Analysis

Five separate linear mixed model analyses were completed using R Studio (version 2025.05.0 + 496) to evaluate whether the CST, listening effort, speech clarity, total impression, and background noise awareness scores differed between conditions of hearing aid program, azimuth, and masker type (fixed effects), with participants as the random effect. Analyses were completed using Type III ANOVA with the significance level set at α = .05. Significance levels for both main and interaction factors and the variance explained in each linear mixed model are presented in Table 2. Post hoc analyses were completed using the lmerTest (version 3.2.0; Kuznetsova et al., 2017) and emmeans (version 2.0.1; Lenth & Piaskowski, 2025) packages with *p*-value and denominator degrees of freedom calculated using the Kenward-Roger method.

**Table 2.** Significance Levels for Both Main and Interaction Factors and the Variance Explained in Each Linear Mixed Model.

Factors	CST	Listening Effort	Speech Clarity	Total Impression	Background Noise Awareness
Variance explained by fixed effects (marginal)	46%	44%	41%	42%	39%
Variance explained by fixed and random effects (conditional)	77%	68%	65%	68%	67%
Intercept	***p* < .001**	***p* < .001**	***p* < .001**	***p* < .001**	***p* < .001**
Azimuth	***p* < .001**	***p* < .01**	***p* < .001**	***p* < .001**	*p* > .05
Program	***p* < .001**	***p* < .001**	***p* < .001**	***p* < .001**	***p* < .001**
Masker	***p* < .001**	***p* < .001**	***p* < .001**	***p* < .001**	***p* < .001**
Program × Masker	***p* < .001**	***p* < .001**	***p* < .01**	***p* < .01**	***p* < .01**
Program × Azimuth	***p* < .001**	***p* < .01**	***p* < .001**	***p* < .001**	***p* < .05**
Azimuth × Masker	***p* < .001**	*p* > .05	***p* < .01**	***p* < .01**	*p* > .05
Program × Azimuth × Masker	***p* = .002**	*p* > .05	*p* > .05	***p* < .01**	*p* > .05

Significant values are bolded.

Model linearity, independence, and homoscedasticity were assessed via graphical plots, Shapiro-Wilk, and Levene's test for homoscedasticity. Correlations among predictors were inspected to evaluate multicollinearity using variance inflation diagnostics. Pairwise comparisons with family-wise Bonferroni corrections were also completed to quantify the magnitude of the observed differences.

The assumptions of normality and lack of outliers were evaluated using Shapiro-Wilk's statistics (Shapiro & Wilk, 1965) and graphical visualization of the residuals. Shapiro-Wilk's normality test was not significant for any linear mixed models (*p* > .05), indicating a normal distribution. All test conditions were randomized; as such, no serial dependence was expected.

Residual-fitted plots indicated mild heteroscedasticity for the CST model and some deviation in the upper fitted range consistent with ceiling effects. On this note, Levene's test indicated heterogeneity of variance in residuals across masker levels, *F*(1, 318) = 16.4, *p* < .001, with higher variability observed for the multitalker babble noise (SD = 12.48) compared to the speech-shaped noise (SD = 8.99). Furthermore, for the speech clarity, and total impression models, Levene's test was significant for the masker factor [*F*(1,318) = 6.28, *p* = .01, *F*(1,318) = 6.11, *p* = .01] with higher variability observed for the multitalker babble noise (SD = 1.38 and 1.33) compared to the speech shaped noise (SD = 1.15 and 1.11) respectively. Levene's test was also significant in the effort model for the azimuth factor *F*(1,318) = 4.22, *p* = .04, with higher variability for the front condition (SD = .88) compared to the side condition (SD = .72). Lastly, Levene's test for homoscedasticity was nonsignificant (*p* > .05) for all the factors in the background noise awareness model.

To assess robustness, we refit the models allowing masker/azimuth-specific residual variances (heteroscedastic specification) and compared estimates to the homoscedastic models. Fixed-effect estimates were unchanged, and the 95% confidence intervals were nearly identical, with standard errors increasing only by approximately 1%, indicating that heteroscedasticity is unlikely to influence statistical inferences (Rosopa et al., 2013).

### Connected Speech Test Results

**Figure 2.**
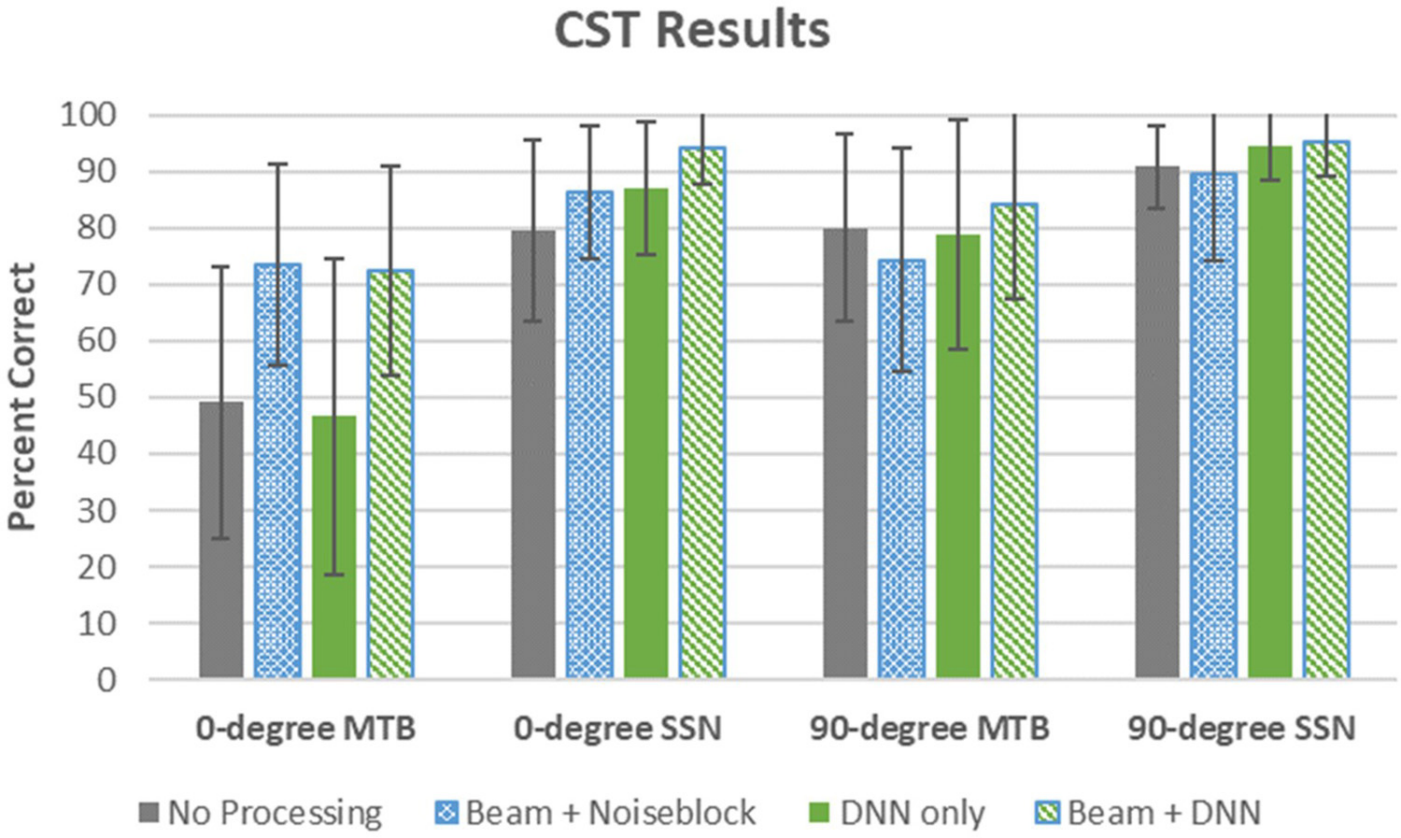
Speech-in-noise recognition results from the CST separated by azimuth (speech from the front 0° or speech from the side 90°) and by noise type (multitalker babble MTB and speech-shaped noise SSN) for the four programs tested. Error bars represent ±1 SD.

Speech-in-noise recognition was evaluated using the CST, and results for the four conditions separated by speech azimuth and noise type are presented in Figure 2.

For statistical analysis, the percentage correct for each CST list was converted to rationalized arcsine units (RAU: Studebaker, 1985) before analysis. Pairwise comparison differences between programs are reported in RAU. The linear mixed model for the CST outcome variable indicated that fixed effects (marginal) accounted for 46% of variance, while both fixed and random effects (participant, conditional) explained 77% of variance in the linear mixed model analysis. The model revealed significant main effects of the fixed factors of azimuth (*F*_[1, 285]_ = 128.92, *p* < .001), hearing aid program (*F*_[3, 285]_ = 23.58, *p* < .001), and masker type (*F*_[1, 285]_ = 335.54, *p* < .001). A three-way interaction was also observed between the factors of azimuth, program, and masker (*F*_[3, 285]_ = 5.16, *p* = .002). Post hoc power simulation for the CST linear mixed model to detect a three-way interaction effect was 94.4% (CI: 92.8%–95.7%)

Pairwise comparisons for the significant main effects of hearing aid program indicated that Beam + DNN had significantly better scores than the other programs, with the largest mean difference observed in comparison to the No Processing program (mean difference = 14.47, *p* < .001) followed by DNN-only (mean difference = 11.41, *p* < .001) then Beam + NB (mean = 7.56, *p* < .001). Beam + NB was also significantly better than the No Processing condition (mean difference 6.89, *p* = .001). As noted in Table 2, there were two-way and three-way interactions between the factors of program, masker, and azimuth. The nature of the three-way interaction effect (*F*_[3, 285]_ = 5.16, *p* = .002) is characterized below.

#### Speech From the Front

Pairwise comparisons indicated that in MTB, programs with directionality (i.e., Beam + DNN and Beam + NB) were significantly better than those programs without directionality (i.e., No Processing and DNN-only), with a reported *p* < .001 for all contrasts. Programs without directionality (No Processing and DNN-only) were not significantly different than one another, nor were the programs with directionality (Beam + DNN vs Beam + NB, *p* *>* .05*)*. The programs with directionality provided an average improvement of 25% in speech in noise recognition compared to the programs without directionality (Figure 2). For speech recognition in SSN, Beam + DNN significantly outperformed the other hearing aid programs. Although the largest difference recorded was relative to No Processing (mean difference = 20.25, *p* < .001), Beam + DNN significantly outperformed the other program with directionality (Beam + NB) with a mean difference of 11.46, *p* *=* .01 and also the program with DNN-only with a mean difference of 10.92, *p* *=* .02.

#### Speech From the Side

Beam + DNN significantly outperformed Beam + NB when the speech was presented in MTB (mean difference = 11.90, *p* = .007). There were no other significant contrasts in this condition. In the SSN, there was no significant difference observed across programs, with good CST performance across the programs, with scores ranging between 89.4% and 95.1% correct.

### Subjective Rating Results

#### Clarity and Total Impression

##### Clarity

The linear mixed model for the clarity outcome variable indicated that fixed effects (marginal) accounted for 41% of variance, while both fixed and random effects (conditional) explained 65% of variance. This model revealed significant main effects of all fixed factors, azimuth (*F*_[1, 285]_ = 40.09, *p* < .001), hearing aid program (*F*_[3, 285]_ = 13.85, *p* < .001), and masker type (*F*_[1, 285]_ = 242.92, *p* < .001). Pairwise comparison for the significant main effects of the hearing aid program indicated that Beam + DNN had significantly better clarity than the other three programs: No Processing (mean difference = 1.36, *p* < .001); DNN-only (mean difference = .82, *p* < .001); Beam + NB (mean difference = .76, *p* = .003). Beam + NB was also significantly better than No Processing (mean difference = .60, *p* = .03). There was also two-way interaction effects observed between the factors of program and masker (*F*_[3, 285]_ = 5.34, *p* *=* .001), program and azimuth (*F*_[3, 285]_ = 6.72, *p* < .001) and azimuth and masker (*F*_[1, 285]_ = 8.94, *p* = .003). Post hoc power simulation for the clarity linear mixed model to detect the two-way interaction effects of program and masker was 94.7% (CI: 93.1%–96.0%), program and azimuth was 97.2% (CI: 96.0%–98.1%), and azimuth and masker was 89.0% (CI: 86.9%–90.9%).

###### Effect of Masker Type

**Figure 3.**
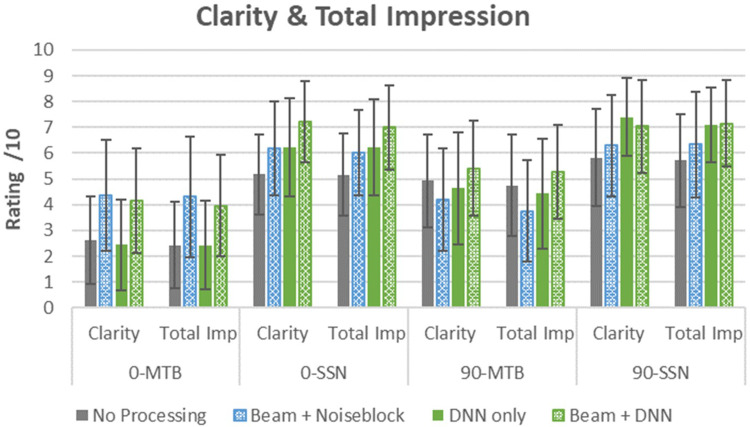
Listener ratings for clarity and total impression (total imp), where a higher score is considered better performance (SSN: speech-shaped noise; MTB: multitalker babble). Error bars represent ±1 SD.

As expected, the clarity ratings were significantly better when in SSN than when in MTB for both azimuths (*p* < .001, Figure 3), pointing to improved hearing aid signal processing benefits for the steady state masker. Collapsed across azimuth, Beam + DNN recorded the highest mean clarity ratings in both masker conditions. In MTB, Beam + DNN was significantly better than both the No Processing program (mean difference = 1.00, *p* = .006) and the DNN-only program (mean difference = 1.24, *p* < .001). In SSN, the Beam + DNN program was significantly better than both the No Processing program (mean difference = 1.71, *p* < .001) and the Beam + NB program (mean difference = 1.01, *p* = .005). Clarity ratings for the DNN-only program were also significantly better than those from the No Processing program (mean difference = 1.31, *p* = < .001).

###### Effect of Azimuth

Collapsed across maskers, programs with directionality had better clarity ratings than programs without directionality for the front azimuth conditions. While Beam + DNN did not differ from the Beam + Noise Block program (*p* *>* .05) when speech was presented from the front, both programs had significantly better clarity ratings than the nondirectional programs (No Processing and Beam + NB, mean difference = 1.33, *p* < .001); No Processing and Beam + DNN (mean difference = 1.79, *p* < .001); DNN-only and Beam + NB (mean difference = .90, *p* = 0.02); DNN-only and Beam + DNN (mean difference = 1.36, *p* < .001). When speech was presented from the side, Beam + DNN had significantly better clarity ratings than programs without DNN (mean difference with No Processing = .92, *p* = .01); mean difference with Beam + NB = 1.01, *p* < .003).

##### Total Impression

The linear mixed model for the total impression ratings indicated that fixed effects (marginal) accounted for 42% of variance, while both fixed and random effects (conditional) explained 68% of variance in the linear mixed model analysis. Similar to the CST outcome, this model revealed significant main effects of the fixed factors of azimuth (*F*_[1, 285]_ = 36.46, *p* < .001), hearing aid program (*F*_[3, 285]_ = 14.23, *p* < .001), and masker type (*F*_[1, 285]_ = 282.34, *p* < .001). Pairwise comparison for the significant main effects of hearing aid program indicated that Beam + DNN had significantly better total impression scores than the other programs, with the largest mean difference observed in comparison to the No Processing program (mean difference = 1.32, *p* < .001), followed by DNN-only (mean difference = 0.79, *p* < .001), and Beam + NB (mean difference = 0.73, *p* = .002). A three-way interaction effect was observed between the factors of azimuth, program, and masker (*F*_[3, 285]_ = 3.86, *p* < .01), the nature of which is described below. The power simulation to detect a three-way interaction effect was 87.5% (CI: 85.3%–89.5%).

###### Speech From the Front

In MTB, pairwise comparisons indicated that programs with directionality had better total impression ratings compared to those that did not (No Processing and Beam + DNN, mean difference = 1.53, *p* < .001); No Processing and Beam + NB (mean difference = 1.88, *p* < .001); DNN-only and Beam + DNN (mean difference = 1.52, *p* = .001); DNN-only and Beam + NB (mean difference = 1.87, *p* < .001). The two programs with directionality (i.e., Beam + DNN and Beam + NB, *p* > .05) did not differ significantly in total impression, nor did the two programs without directionality (i.e., No Processing and DNN, *p* > .05). In SSN, total impression ratings from the Beam + DNN program were significantly better than those for the No Processing program (mean difference = 1.84, *p* < .001).

###### Speech From the Side

In the MTB condition, Beam + DNN was rated significantly better for total impression than the Beam + NB program (mean difference = 1.51, *p* = .002). In SSN, the programs that incorporated DNN were rated significantly better for the total impression than the No Processing program (DNN-only, mean difference = 1.38, *p* *=* .005); Beam + DNN (mean difference = 1.41, *p* = .004)).

#### Listening Effort and Background Noise Awareness

##### Listening Effort

The linear mixed model for listening effort ratings indicated that fixed effects (marginal) accounted for 44% of variance, while both fixed and random effects (conditional) explained 68% of variance in the linear mixed model analysis. Similar to both the speech recognition and total impression, this model revealed significant main effects of all fixed factors (*p* < .01).

Pairwise comparison for the significant main effects of the hearing aid program indicated that Beam + DNN had significantly less listening effort than the other programs, with the largest mean difference observed in comparison to the No Processing program (mean difference = 0.98, *p* < .001). There was a significant improvement for the Beam + DNN versus Beam + NB (mean difference = 0.52, *p* < .001) and Beam + DNN and DNN-only (mean difference 0.56, *p* = < .001). There were also significant improvements for Beam + NB versus No Processing (mean difference = 0.46, *p* = .004) and for DNN-only versus No Processing (mean difference = 0.43, *p* = .001).

Two-way interactions were observed between the factors of program and masker (*F*_[3, 285]_ = 6.24, *p* < .001) and between program and azimuth (*F*_[3, 285]_ = 5.30, *p* < .001). The power simulation for this model to detect the two-way interaction effects of program and masker was 97.5% (CI: 96.3%–98.4%), and program and azimuth was 94.0% (CI: 92.1%–95.2%).

###### Listening Effort in MTB

**Figure 4.**
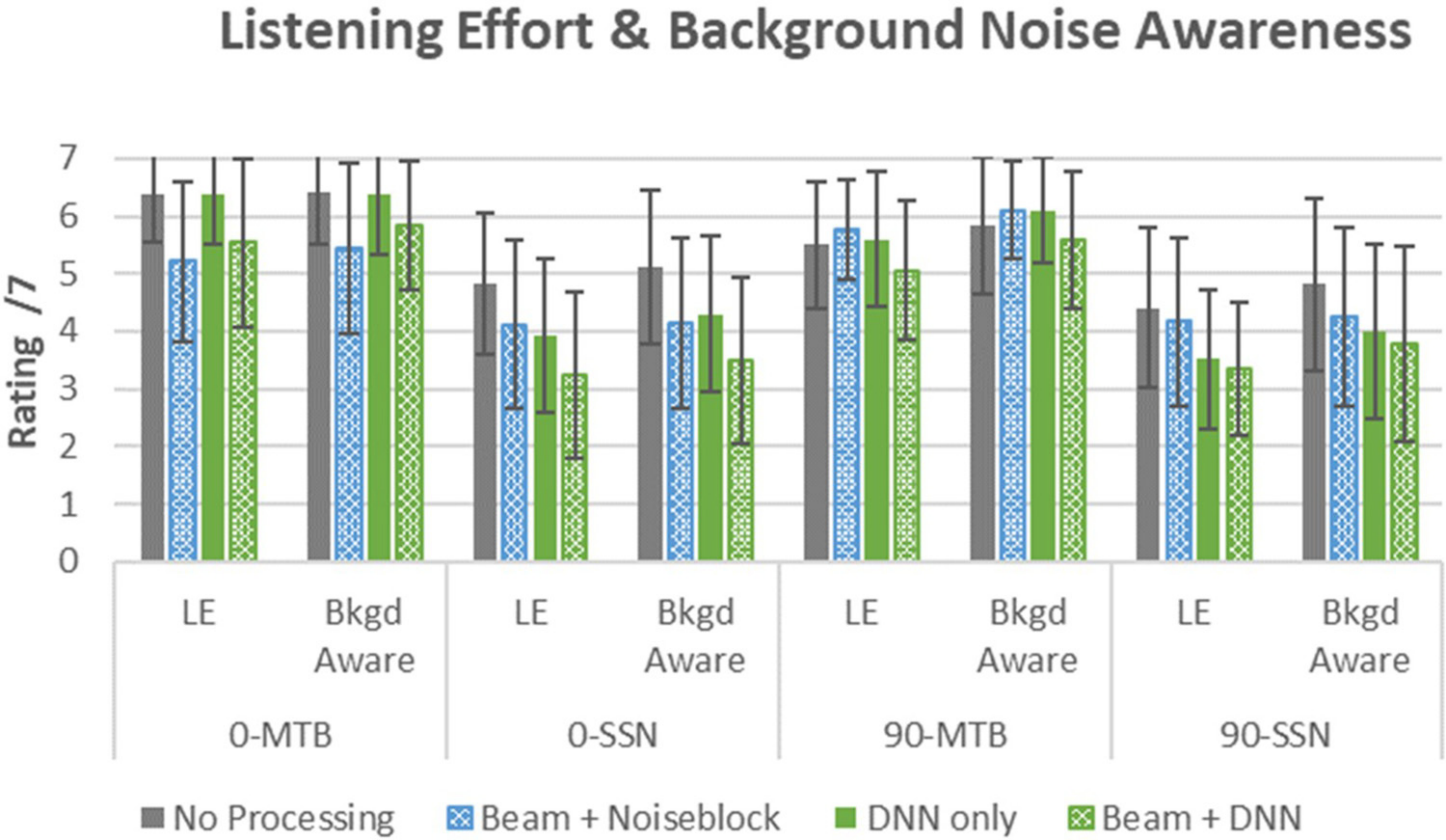
Listener ratings of listening effort (LE) and background noise awareness (BKGD aware). Lower scores indicate better performance (SSN: speech-shaped noise; MTB: multitalker babble; DNN: deep neural network). Error bars represent ±1 SD.

As seen in Figure 4, there were directional benefits observed for the front condition in MTB in comparison to the omnidirectional programs. Combined across azimuth, pairwise comparisons corrections indicated that Beam + DNN had the lowest effort rating with a significant difference recorded relative to the No Processing program (mean difference = 0.65, *p* = .004) and DNN-only (mean difference = 0.70, *p* < .002). No other contrasts were significant.

##### Listening Effort in SSN

In SSN, Beam + DNN again had the best listening effort scores with significant improvement recorded relative to the programs that did not employ DNN: No Processing (mean difference = 1.32, *p* < .001), and Beam + NB-only (mean difference = 0.85, *p* < .001). There was also a significant improvement in listening effort for DNN-only versus No Processing (mean difference = 0.90, *p* < .001).

###### Listening Effort With Speech From the Front

With speech presented from the front, the programs with directionality were rated significantly better for listening effort when compared to the No Processing condition: Beam + DNN programs (mean difference = 1.21, *p* < .001), and Beam + Noise Block (mean difference = 0.93, *p* < .001). Further, the combination of Beam + DNN resulted in significantly less listening effort than the DNN-only condition (mean difference = 0.76, *p* < .001).

###### Listening Effort With Speech From the Side

For the side condition, Beam + DNN had the lowest listening effort ratings and was significantly better than both programs that did not use DNN processing (*p* < .001).

##### Background Noise Awareness

The linear mixed model for the background noise awareness outcome also indicated that fixed effects (marginal) accounted for 39% of variance, while both fixed and random effects (conditional) explained 67% of variance in the linear mixed model analysis. This model revealed significant main effects of the fixed factors hearing aid program (*F*_[3, 285]_ = 14.65, *p* < .001), and masker type (*F*_[1, 285]_ = 305.40, *p* < .001).

Pairwise comparison for the significant main effects of the hearing aid program indicated that Beam + DNN had significantly lower (better) scores for background noise awareness than No Processing (mean difference = 0.91, *p* < .001) and DNN-only (mean difference = 0.55, *p* < .001). There was also a significant reduction in background noise awareness over No Processing by the Beam + NB program (mean difference = 0.57, *p* < .001).

Similar to listening effort, a two-way interaction effect was observed between the factors of program and masker (*F*_[3, 285]_ = 5.47, *p* < .01) and program and azimuth (*F*_[3, 285]_ = 3.15, *p* = .03). Post hoc power simulation for this model to detect the two-way interaction effects of program and masker was 94.3% (CI: 92.7%–95.7%), and program and azimuth was 76.7% (CI: 74.0%–79.3%).

###### Effects of Background Noise Type

In MTB, pairwise comparisons indicated that there was no significant difference between programs (*p* > .05). In the SSN condition, all hearing aid programs were significantly different, except for the Beam + NB versus DNN-only. The largest difference for this condition was observed between No Processing and Beam + DNN (mean difference = 1.41, *p* < .001), favoring the latter program. Beam + DNN was also significantly better than Beam + NB (mean difference = 0.63, *p* = .01) and also DNN-only (mean difference = 0.58, *p* = .03). Beam + NB received significantly better ratings for background noise awareness than No Processing (mean difference = 0.78, *p* < .001).

###### Azimuth Effects and Background Noise Awareness

Collapsed across masker type, post hoc comparisons indicated that when the speech was presented from the front, programs that used directionality were rated significantly better for background noise awareness than the ones that did not. The largest difference in score was observed between No Processing and Beam + DNN (mean difference = 1.10, *p* < .001); followed by No Processing and Beam + NB (mean difference = 0.98, *p* < .001); DNN-only and Beam + DNN (mean difference = 0.66, *p* = .007); and, DNN-only and Beam + NB (mean difference = 0.54, *p* < .05), all favoring the programs with Beam.

When speech was presented from the side, Beam + DNN was rated significantly better for background noise awareness than the programs that did not use DNN technology: No Processing and Beam + DNN (mean difference = 0.73, *p* *=* *.*002) and Beam + NB and Beam + DNN (mean difference = 0.57, *p* = .03).

#### Comparison Across Outcomes

**Figure 5.**
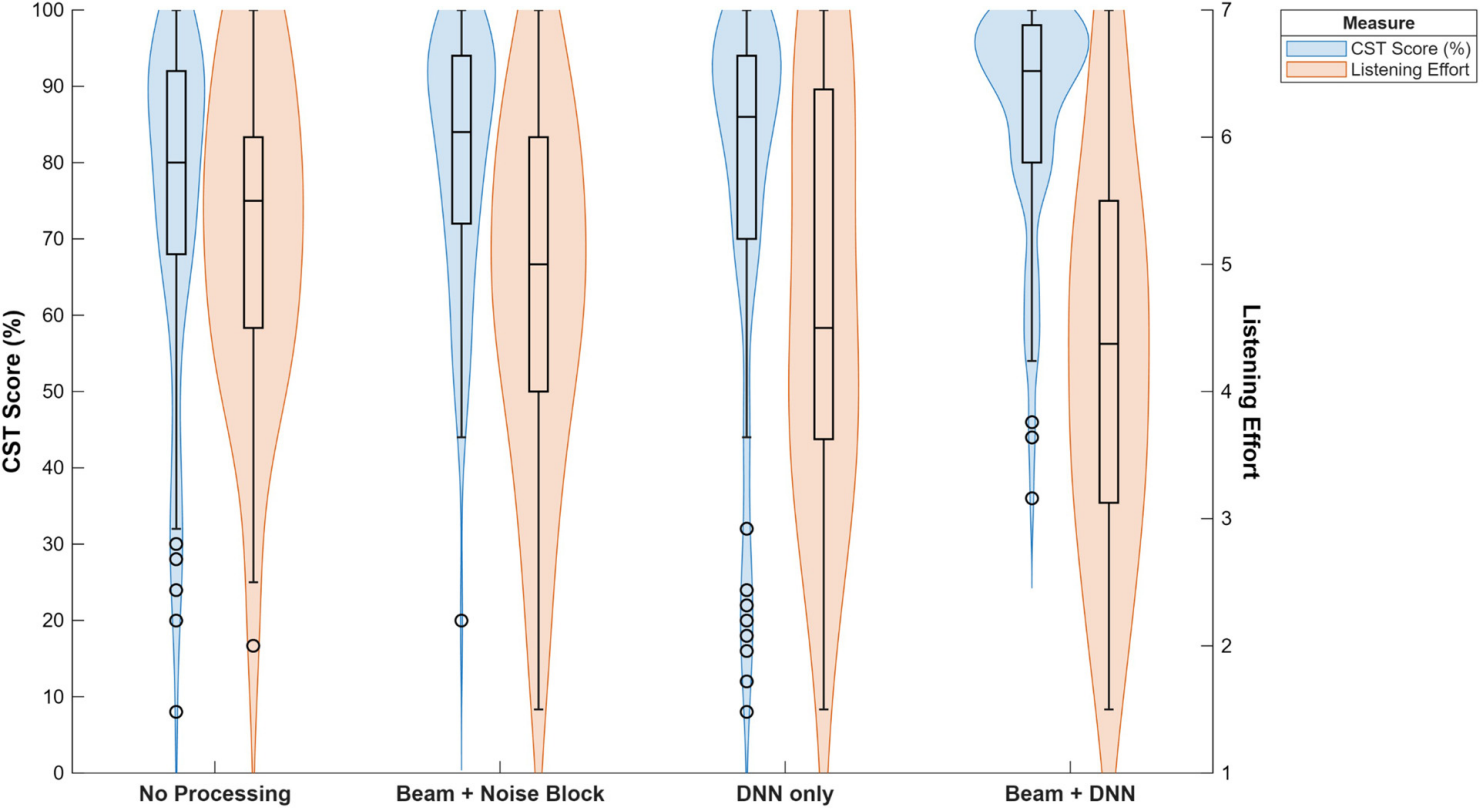
The overall effects of the program on speech recognition on the percent correct score on the connected speech test (CST) and listening effort ratings. In this figure, we see that the Beam + DNN has both the highest speech recognition and the lowest listening effort across conditions.

When considering the analysis averaged over the levels of azimuth and masker, the Beam + DNN program demonstrated significantly better speech recognition (*p* < .001), sound quality ratings (clarity and total impression, *p* < .001), and less listening effort (*p* < .01) scores compared to the other hearing aid programs. Figure 5 provides a visual example of the benefit of Beam + DNN across a pair of factors that affect successful communication in background noise, with both an improvement in speech recognition and a reduction of listening effort.

#### Predictors of Total Impression Rating

We also examined whether CST scores, listening effort, speech clarity, and noise awareness predicted the total impression scores. The ratings of these predictors were repeated within participants in this study; as such, they were accounted for in the model for within- and between-subject comparisons. We also included in the model the factors of program, azimuth, and masker. While model diagnostics indicated no evidence of nonlinearity (Shapiro-Wilk, *p* > .05), multicollinearity (variance inflation factor <5), or heteroscedasticity (Levene's test *p* > .05), graphical visualization of the data indicated mild deviation in the lower tail.

Within-subject increases in CST scores (β = .19, SE = .06), speech clarity (β = 1.41, SE = .05), and better listening effort (β = −.37, SE = .08) were associated with significantly (*p* < .001) increased total impression ratings, when accounting for the factors of program, azimuth, and masker.

## Discussion

In this study, the primary outcome of speech recognition in steady state and multitalker babble was evaluated with a group of older adults fitted with Phonak Audéo I90-Sphere hearing aids in four settings that varied in combinations of microphone (Omni and Directional) and noise reduction strategies (off, Noise Block, DNN). Clinically typical venting was used in the fitting of the receiver domes. Ratings of sound quality, listening effort, and background noise awareness were collected as secondary outcome measures in the same conditions. Passages from the Connected Speech Test were presented at 70 dB SPL at 0 dB SNR, which would be considered a challenging but realistic listening environment.

With this test battery of both subjective and objective outcomes, we found that DNN + Beam consistently outperformed the other settings across the five metrics evaluated: speech intelligibility (especially in SSN); clarity and total impression ratings (highest ratings); and listening effort and background noise awareness (lowest ratings). We were more likely to measure improvements for this DNN processor when: (a) it was combined with directionality/beamforming; (b) when the background noise was SSN; and (c) when the speech signal was presented from the side. These trends in the reported results appear in different combinations across the outcomes reported above, but in general, most listening situations demonstrated some benefit for the hearing aid processing condition that provided DNN with directionality.

Outcomes were influenced by both noise type and spatial configuration. Listening in multitalker babble was more challenging than in steady-state noise, and listening in spatially separated speech and noise was more difficult when speech originates from the front compared to the side. These findings are consistent with numerous past hearing device studies (e.g., Mauger et al., 2012; Picou & Ricketts, 2019; Schoof & Rosen, 2014; Vermiglio, 2019), and this reflects the goal of assessing the DNN in challenging listening scenarios that relate to the factors of difficult listening in noise.

Overall, these results show that improvements in speech recognition tend to relate to improved clarity and overall impression, less listening effort, and a reduction in background noise awareness. Speech recognition scores at a challenging 0 dB SNR exhibited ceiling effects for some conditions, while subjective outcomes did not. In these cases, despite the high speech recognition scores across all hearing aid conditions, the reduced listening effort, decreased awareness of background noise, and better sound quality ratings suggest that listeners experienced additional perceptual benefits that have been found to support successful communication in group settings (Nicoras et al., 2023).

There were also a number of conditions that used directionality in combination with non-DNN noise reduction that provided similar performance. As noted in Table 1, the Beam + DNN program uses an adaptive monaural beamformer where each hearing aid creates a directional pattern using its own microphones, whereas the Beam + NB program uses binaural beamforming, combining input from the four microphones across the linked left and right hearing aids. Regardless of these differences, the results from this study suggest that directionality and traditional noise reduction, as well as directionality with DNN denoising, provided noise management benefits, but with scenario-specific differences. For example, in the reduction of awareness of background noise, directionality played a key role with Beam + DNN and Beam + NB receiving the most favorable ratings when collapsed across all conditions. However, there were advantages of the Beam + DNN over and above the Beam + NB when the noise type and azimuth of the speech were considered. While directionality was the driving force behind the better ratings in the front-facing conditions, when listening to speech from the side and when listening in SSN, Beam + DNN was rated significantly better than Beam + NB.

Throughout the results, we see an interaction between the features of directionality and the noise reduction technologies tested in this study. While the DNN technology was not as effective with the MTB as it was with the SSN, its implementation with beamforming continued to provide objective and subjective benefit in this more difficult masker, particularly when speech was coming from the side. With the MTB consisting of six overlapping talkers at the same level as the target, the DNN may have detected segments of intelligible speech within the MTB and may have treated it like the target. We do not have a moment-by-moment analysis of the DNN's behavior in this dataset with which to evaluate this speculation. However, if this is true, the results are consistent with the anticipated effect: when active, the directional microphones reduce the level of the nonfrontal MTB, which in turn supports the DNN's ability to denoise the remaining signal. This is consistent with the principles of modern hearing aid noise reduction discussed by Andersen et al. (2021), where beamforming and postfiltering (in this case, a neural network trained for postfiltering) are considered core principles of noise reduction, and the two can have co-dependent effects. By using both of these sequentially, one can exploit the typical spatial separation of the target and maskers first to clean the signal prior to DNN. This may increase the input SNR to the DNN, which may, in turn, support better performance from the DNN at lower signal-to-noise ratios, observed both here and in our previous study (Ashkanichenarlogh et al., 2025). These results provide an update to a recent systematic review (Lakshmi et al., 2021), which reviewed digital noise reduction technology studies from 2000 to 2017, and found no significant improvement to speech intelligibility, whether the technologies were or were not combined with directionality.

This combination has the potential to offer auditory benefit in a wider range of complex communication situations compared to the Beamformer or DNN-only strategy. This has clinical implications because the DNN with beamformer offered the best overall performance when considering both speech recognition and listening effort together, compared to the next-best processing combination of noise reduction and beamforming. This presents an additional type of noise management for use in hearing aids, allowing hearing aid users to apply a new strategy when in noisy environments. Additionally, this study contributes to the growing body of DNN evidence by including MTB and two target-speech azimuths, both of which highlight the benefit of multiple synergistic noise reduction strategies for commercial hearing aids.

## Limitations

The presentation level of 70 dB SPL at 0 dB SNR was chosen to evaluate the DNN in a challenging but ecologically valid condition. However, this high presentation level, along with well-fitted hearing aids, may have resulted in the ceiling effect in speech recognition in SSN. This ceiling was less apparent in the self-rating measures, such as listening effort. This highlights the importance of incorporating measures that are sensitive to a range of effects, particularly when testing in a condition that is ecologically valid, which may place speech intelligibility scores at ceiling performance. Further, the type of beamforming available within the tested hearing aids was not identical between the noise reduction and DNN conditions: this has the advantage of representing the actual clinical settings of the hearing aids under evaluation, but does not provide the same beamforming type across conditions. This limitation should be considered when interpreting the results presented. Lastly, the post hoc analysis for background noise awareness was underpowered for the two-way interaction effect of program and azimuth; as such, these results are to be interpreted with caution.

### Future Directions

The results of this study indicated that the DNN's ability to improve speech perception was affected by noise type. Specifically, the DNN performance was better in SSN than with MTB with two talkers per location, which provided a more intelligible masker intended to challenge the DNN system. Without beamforming, the DNN alone was less effective with this masker type, at least within the lab environment. Whether and how this result would relate to real-world test environments, or whether further development of DNN strategies may be possible, would require further study and innovation. For example, future directions for algorithm development might be to further train the DNN to better identify target speech versus competing background babble. In addition, the tested DNN reduced awareness of background noise, which was associated with improved speech recognition in noise, less listening effort, and higher speech clarity and impression. However, we did not assess whether the reduction of awareness of background noise was preferred. Future studies could measure preference for background noise reduction in real-world situations to better understand the relationships between background noise reduction and environmental awareness.
